# Healthy Lifestyle and the Likelihood of Becoming a Centenarian

**DOI:** 10.1001/jamanetworkopen.2024.17931

**Published:** 2024-06-20

**Authors:** Yaqi Li, Kaiyue Wang, Guliyeerke Jigeer, Gordon Jensen, Katherine L. Tucker, Yuebin Lv, Xiaoming Shi, Xiang Gao

**Affiliations:** 1 Department of Nutrition and Food Hygiene, School of Public Health, Institute of Nutrition, Fudan University, Shanghai, China; 2 Department of Medicine, Larner College of Medicine, University of Vermont, Burlington; 3 Department of Biomedical and Nutritional Sciences, University of Massachusetts Lowell; 4 National Institute of Environmental Health, Chinese Center for Disease Control and Prevention, Beijing, China

## Abstract

**Importance:**

Previous studies have reported that lifestyle factors were associated with life expectancy and/or mortality, but most of them studied the middle-aged or older age groups (aged ≥60 years), and few focused on people aged 80 years or older.

**Objectives:**

To examine healthy lifestyle and the likelihood of becoming centenarians among people aged 80 years or older in China.

**Design, Settings, and Participants:**

Using data from the Chinese Longitudinal Healthy Longevity Survey, a nationally representative and one of the largest prospective cohorts targeting people aged 80 years or older established in 1998, a community-based, prospective nested case-control study was performed. Data were analyzed from December 1, 2022, to April 15, 2024.

**Exposures:**

A healthy lifestyle score for 100 (HLS-100, ranging from 0 to 6), including smoking, exercise, and dietary diversity, was constructed, with higher scores indicating potentially better health outcomes.

**Main Outcomes and Measures:**

The primary outcome was survivorship to becoming a centenarian by 2018 (the end of follow-up). Information on sociodemographic characteristics, lifestyle factors, and other covariates was collected.

**Results:**

The sample comprised 5222 individuals (61.7% women, mean [SD] age, 94.3 [3.3] years), including 1454 identified centenarians and 3768 controls (died before becoming centenarians) matched by age, sex, and year of entry. During a median follow-up of 5 (IQR, 3-7) years, 373 of 1486 individuals among the lowest HLS-100 (0-2) group and 276 of 851 individuals among the highest HLS-100 (5-6) group became centenarians. The adjusted odds ratio (AOR) comparing the highest vs the lowest HLS-100 groups was 1.61 (95% CI, 1.32-1.96; *P* < .001 for trend). An association was noted when we further treated centenarians with relatively healthy status as the outcome, as evaluated by self-reported chronic conditions, physical and cognitive function, and mental wellness (AOR, 1.54; 95% CI, 1.05-2.26). Similar results were observed in other sensitivity analyses.

**Conclusions and Relevance:**

In this case-control study of Chinese older adults, adhering to a healthy lifestyle appears to be important even at late ages, suggesting that constructing strategic plans to improve lifestyle behaviors among all older adults may play a key role in promoting healthy aging and longevity.

## Key Points

**Question:**

Is a healthy lifestyle associated with a higher likelihood of becoming a centenarian?

**Findings:**

In this nested case-control study, individuals aged 80 years or older were evaluated, including 1454 centenarians and 3768 individuals who died before reaching 100 years. Individuals with the highest healthy lifestyle score (constructed from smoking, exercise, and dietary diversity) had a significantly higher likelihood of becoming a centenarian, compared with those with the least healthy lifestyle behaviors.

**Meaning:**

The findings of this study suggest that adhering to a healthy lifestyle could be beneficial even at a very advanced age.

## Teaser

This nested case-control study examines the factors associated with individuals aged 80 years or older becoming centenarians.

## Introduction

With major progress in social, economic, and medical development, life expectancy at birth increased substantially in recent decades and was estimated at 73.5 years globally and 77.6 years in the mainland of China in 2019.^1,2^ Parallel with the increased life expectancy, the aging population has been rapidly expanding, raising the public health challenge of promoting healthy aging and longevity.

In addition to sociodemographic and genetic determinants, several modifiable lifestyle factors are important for healthy aging and longevity. These have been associated with aging-related outcomes such as cognitive performance and life expectancy.^3,4,5,6^ However, most existing studies have targeted a broad spectrum of middle-aged (≥45 years) and/or older (≥60 years) groups,^7,8,9^ with limited understanding of how lifestyle factors would affect health outcomes in individuals of advanced age (≥80 years).^10,11^ As the number of centenarians continued to increase,^12^ there have been accumulating observations of compression of morbidity and/or disease-related disability in those individuals,^13,14^ suggesting that “the older an individual gets, the healthier he or she has been, at least in relation to those still at younger ages.”^13^ In this context, studying the potential lifestyle factors associated with survivorship to become a centenarian among those already members of the very advanced age group can provide unique insight into achieving healthy longevity and generate evidence-based strategies to promote healthy aging.

Therefore, using data from the Chinese Longitudinal Healthy Longevity Survey (CLHLS), a nationally representative cohort of the older population, we adopted a nested case-control study design, with identified centenarians as cases, to prospectively investigate the association between modifiable lifestyle factors and likelihood of becoming a centenarian in individuals aged 80 years and older.

## Methods

### Study Design and Population

The CLHLS is a nationwide, ongoing survey that randomly sampled half of the counties and cities in 22 of the 31 provinces in mainland China, covering approximately 85% of the total population.^15^ The CLHLS collected data from 8 waves of surveys conducted in 1998, 2000, 2002, 2005, 2008, 2011, 2014, and 2018, with follow-ups of preexisting participants and recruitment of new participants in each round of the survey; details of the CLHLS can be found elsewhere.^16,17^ Ethical approval was obtained from the Biomedical Ethics Committee of Peking University, and all the participants or their proxy respondents signed the informed consent form; no financial compensation was provided. We followed the Strengthening the Reporting of Observational Studies in Epidemiology (STROBE) reporting guideline for case-control studies.

With our prospective nested case-control design, we first included 17 917 eligible participants (age ≥80 years) who entered the cohort during the first 5 waves of the surveys (1998-2008) and had the potential to age to 100 years or older as of 2018 (the end of follow-up), providing a minimum potential follow-up period of 10 years until 2018. Briefly, for those who entered in 1998, as long as they were 80 years or older at entry, they could potentially live to 100 years in 2018, but for those entered in 2008, they needed to be 90 years or older at entry to become centenarians in 2018 (eFigure in Supplement 1). We then excluded participants who were lost to follow-up or with incomplete information on required lifestyle factors at baseline. Among eligible participants, we identified centenarians as cases and matched them with those who died before reaching age 100 years as controls, with sex, entry year, and age (±1 year) at the year of entry as matching variables, using the method described before.^18^ Cases and controls were matched up to a 1:4 ratio, leaving 5222 participants in our final analysis. The accuracy of the age reported in this cohort and overall data quality have been verified and confirmed by previous studies.^19,20^

### Construction of Healthy Lifestyle Score

We initially constructed a healthy lifestyle score (HLS) with 5 traditional healthy lifestyle components, including smoking, alcohol use, exercise, dietary diversity, and body mass index (BMI). Each lifestyle component was classified as a ternary variable and reencoded as 0, 1, and 2, with higher scores indicating potentially better health outcomes, as described in previous literature.^21,22^ These were then summed to a healthy lifestyle score, ranging from 0 to 10,^21^ as detailed in eTable 1 in Supplement 1. These variables were assessed via questionnaires and physical examinations through a face-to-face interview by trained field workers from the local Centers for Disease Control and Prevention in each survey, as described previously.^17,23^ Later, based on the results from our primary analysis, we reconstructed a new version of the HLS tailored for adults aged 80 years or older, which could better reflect their healthy lifestyle behaviors and related health outcomes, called HLS for 100 (HLS-100), meaning living to 100 years, in which BMI and alcohol use were removed. Lifestyle factors were assessed at baseline and during follow-up surveys. For the primary analysis, we used the lifestyle information collected at baseline to calculate the HLS and HLS-100. A detailed description regarding individual lifestyle factor assessment is provided in the eMethods in Supplement 1.

### Assessment of Covariates

Sociodemographic factors, including age, sex, residence (urban dwellers, rural dwellers), years of education (0, 1-9, >9 years), marital status (married, not married), and self-reported history of chronic conditions in or before the interview, including hypertension (yes, no), diabetes (yes, no), cardiovascular disease (CVD) (yes, no), and cancer (yes, no), were obtained by a face-to-face interview via a questionnaire by well-trained interviewers from the local Centers for Disease Control and Prevention.^15,16^

### Ascertainment of Death

Interviews of a close family member of the participants who had died who were interviewed in the previous wave but died before the subsequent survey and information about the date and cause of death were collected. Date and cause of death were ascertained from official death certificates, and another confirmation was usually needed from the local neighborhood committee if the death certificate was not available, as detailed elsewhere.^24^

### Statistical Analysis

Multivariable conditional logistic regression models were adopted to evaluate the association between the original HLS and living to be a centenarian, odds ratios (ORs) and 95% CIs were estimated and adjusted for sociodemographic factors (residence, years of education, and marital status) and self-reported chronic conditions (hypertension, diabetes, cardiovascular disease, and cancer). We also calculated the OR and 95% CI per-unit increase in the HLS, treating it as a continuous variable considering its *P* value as the *P* value for trend. The association between individual lifestyle component and living to be a centenarian was further investigated, with the other lifestyle factors mutually adjusted. Later, with the recalculated HLS-100, we reexamined healthy lifestyle and likelihood of becoming centenarians. Additionally, we conducted secondary analyses including categorizing lifestyle factors as binary variables, constructing predictive models to estimate the possibility of becoming centenarians, and subgroup and mediation analysis; details are described in the eMethods in Supplement 1.

Several sensitivity analyses were performed. First, we conducted a 2-year lag analysis and a 5-year lag analysis by excluding individuals who lived to 100 years within 2 and 5 years (median follow-up duration of cases) and their matched controls. Second, given the potential benefits of moderate alcohol consumption for some health-related outcomes in the aging population,^25^ we redefined drinking status based on alcohol consumption amount (eMethods in Supplement 1)^26,27^ after excluding those with missing information on alcohol consumption (n = 1417). Third, to consider that lifestyle factors may have unequal associations with the outcome, we recalculated a weighted standardized healthy lifestyle score based on the β coefficient of each lifestyle component in the multivariable conditional logistic regression model. Fourth, we calculated an updated HLS-100 score based on the most recent lifestyle information collected before the end point event (death for controls and reaching age 100 years for cases) and examined its association with the survivorship of centenarians. Fifth, we used becoming centenarians with a relatively healthy status as an outcome, with the health status based on the number of healthy aging indicators, including no self-reported chronic conditions, normal physical and cognitive function, and good mental wellness (eMethods in Supplement 1), ranging from 0 (worst) to 4 (best),^28,29^ and we considered the score greater than or equal to 3 as a relatively healthy status. Sixth, to further examine the results we observed in the nested case-control study design, we applied a Cox proportional hazards regression analysis to the eligible cohort population (n = 10 778) to examine whether there was an association between HLS-100 and the likelihood of becoming centenarians and computed hazard ratios with 95% CIs. Seventh, to address the potential impact of missing covariate data, we excluded individuals with missing covariates at baseline. Eighth, to understand whether the observed association between the overall lifestyle and likelihood of becoming a centenarian was associated with a given lifestyle component, we reconstructed healthy lifestyle scores by excluding each 1 of the 5 lifestyle components at a time and treating it as a confounder in the model.

Statistical analysis was performed using SAS, version 9.4 (SAS Institute Inc) and R, version 4.2 (R Foundation for Statistical Computing). Data were analyzed from December 1, 2022, to April 15, 2024. A 2-sided value of *P* < .05 indicated statistical significance.

## Results

A total of 5222 participants, including 1454 identified centenarians and their matched 3768 controls (died before reaching age 100 years and matched by age, sex, and year of entry), were included in this study. Of this sample, 3223 (61.7%) were women and 1999 (38.3%) were men, with a mean (SD) age of 94.3 (3.3) years. Characteristics of participants at baseline are presented in Table 1. No significant differences were detected between cases (centenarians) and controls (noncentenarians) regarding their sociodemographic characteristics and medical conditions (Table 1).

**Table 1.** Baseline Characteristics of Participants

Characteristic	No. (%)	
	Centenarians (n = 1454)	Noncentenarians (n = 3768)
Age, mean (SD), y	94.5 (3.4)	94.1 (2.0)
Sex		
Women	919 (63.2)	2304 (61.1)
Men	535 (36.8)	1464 (38.9)
Residence, urban dwellers	890 (61.2)	2259 (60.0)
Duration of education, y		
0	1062 (73.0)	2713 (72.0)
1-9	334 (23.0)	939 (24.9)
>9	54 (3.7)	99 (2.6)
Missing	4 (0.3)	17 (0.5)
Married	177 (12.2)	471 (12.5)
BMI	19.1 (4.1)	18.7 (2.6)
Self-reported hypertension	186 (12.8)	486 (12.9)
Self-reported diabetes	8 (0.6)	30 (0.8)
Self-reported cardiovascular disease	40 (2.8)	152 (4.0)
Self-reported cancer	1 (0.1)	16 (0.4)

Abbreviation: BMI, body mass index (calculated as weight in kilograms divided by height in meters squared).

The higher HLS was associated with a higher likelihood of becoming a centenarian in a dose-response manner (*P* < .001 for trend) (Figure). The adjusted OR (AOR) for the highest (8-10) vs the lowest (0-5) HLS groups was 1.33 (95% CI, 1.10-1.62) (Figure). Among individual lifestyle components, never smoking, exercise, and greater dietary diversity were significantly associated with higher odds of becoming a centenarian, whereas no significant association was detected for alcohol use or BMI (eTable 2 in Supplement 1). Therefore, we reconstructed the HLS-100 to include only smoking status, exercise, and dietary diversity, and reconducted the analysis using the HLS-100. The distribution of the number of participants across different healthy lifestyle scores is reported in eTable 3 in Supplement 1. As expected, a similar association between HLS-100 and survivorship of becoming a centenarian was observed with an even higher estimated AOR of 1.61 (95% CI, 1.32-1.96), and all 3 elements of healthy lifestyle behaviors were associated with the likelihood of reaching age 100 years (Table 2). In alignment with the OR we reported, a higher HLS-100 was associated with a greater possibility of becoming centenarians; similar results were observed when computing the possibility according to different category of smoking, exercise, and dietary diversity (eTable 4 in Supplement 1). By categorizing each lifestyle component as a binary variable, we constructed the binary version HLS-100. Similar significant results were observed (eTable 5 in Supplement 1).

**Figure.**
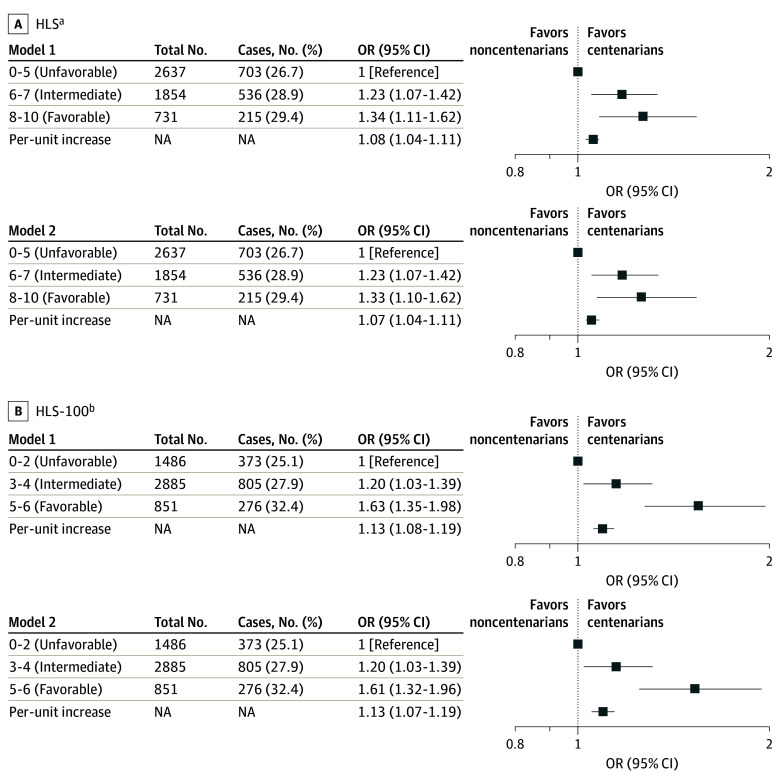
Association Between Healthy Lifestyle Score (HLS) and Becoming a Centenarian Model 1: Crude model. Model 2 for HLS: adjusted for residence (urban dwellers, rural dwellers, and missing), duration of education (0, 1-9, >9 years, and missing), marital status (married, not married, and missing), hypertension (yes, no, and missing), diabetes (yes, no, and missing), cardiovascular disease (yes, no, and missing), cancer (yes, no, and missing). Model 2 for HLS-100: additionally adjusted for alcohol use status (never, former, and current), and body mass index (<18.5, 18.5-23.9, and ≥24.0 [calculated as weight in kilograms divided by height in meters squared]). NA indicates not applicable. ^a^Traditional HLS, composed of smoking status, alcohol use status, exercise status, dietary diversity score, and body mass index. ^b^HLS for living to 100 years, composed of smoking status, exercise status, and dietary diversity score.

**Table 2.** Associations Between Lifestyle Components and Becoming a Centenarian

Lifestyle component	**Cases, No./total No. (%)**	OR (95% CI)^a^
**Smoking status**		
Current (unfavorable)	194/787 (24.7)	1 [Reference]
Former (intermediate)	160/677 (23.6)	0.94 (0.73-1.22)
Never (favorable)	1100/3758 (29.3)	1.25 (1.02-1.53)
Per-unit increase	NA	1.14 (1.03-1.26)
**Exercise status**		
Never (unfavorable)	966/3541 (27.3)	1 [Reference]
Former (intermediate)	91/388 (23.5)	0.80 (0.61-1.04)
Current (favorable)	397/1293 (30.7)	1.31 (1.13-1.53)
Per-unit increase	NA	1.13 (1.05-1.22)
**Dietary diversity score** ^b^		
0-3 (unfavorable)	275/1014 (27.1)	1 [Reference]
4-6 (intermediate)	863/3122 (27.6)	1.09 (0.92-1.29)
7-10 (favorable)	316/1086 (29.1)	1.23 (1.00-1.52)
Per-unit increase	NA	1.11 (1.00-1.23)

Abbreviations: NA, not applicable; OR, odds ratio.

^a^ Adjusted for residence (urban dwellers, rural dwellers, missing), duration of education (0, 1-9, >9 years, missing), marital status (married, not married, missing), hypertension (yes, no, missing), diabetes (yes, no, missing), cardiovascular disease (yes, no, missing), cancer (yes, no, missing), alcohol use status (never, former, and current), and body mass index (<18.5, 18.5-23.9, and ≥24.0 [calculated as weight in kilograms divided by height in meters squared]). The 3 lifestyle components mutually adjusted for each other.

^b^ Dietary diversity was evaluated based on the frequency of consuming 5 food groups: fruits, vegetables, fish, beans, and tea. Participants reporting almost every day, except winter; sometimes or occasionally; or rarely or never for consuming each food item were separately assigned scores of 2, 1, or 0, and the total score of dietary diversity ranged from 0 to 10. Then, scores of 7 to 10 were classified as favorable (2), 4 to 6 as intermediate (1), and 0 to 3 as unfavorable (0).

No interactions between healthy lifestyle score and residence, years of education, marital status, chronic conditions, alcohol use status, and BMI were found (*P* > .05 for interaction for all) (eTable 6 in Supplement 1). Although updated chronic conditions explained 34.8% of the association between HLS-100 and the survivorship of becoming centenarians, no statistical significance was detected (95% CI, −1.9% to 71.5%; *P* = .06) (eTable 7 in Supplement 1).

Robustness of the observed association was confirmed in sensitivity analyses (Table 3). Median follow-up was 5 (IQR, 3-7) years; 373 of 1486 individuals among the lowest HLS-100 (0-2) group and 276 of 851 individuals among the highest HLS-100 (5-6) group became centenarians. After excluding centenarians who were followed up for less than 2 years and their corresponding controls, the association remained significant (AOR, 1.58; 95% CI, 1.28-1.96), and further exclusion of cases with follow-up time of less than 5 years showed a similar result (AOR, 1.55; 95% CI, 1.19-2.02), the likelihood of becoming centenarians regarding per healthy lifestyle score increase was stable. Similar patterns were observed when using the redefined HLS-100 developed by weighted score for each lifestyle factor (value of β coefficient can be found in eTable 8 in Supplement 1), the updated HLS-100 calculated from the most recent lifestyle information collected before end point events, and the cohort study design with Cox proportional hazards regression analysis, excluding participants with missing covariates. Specifically, when restricting becoming centenarians with a relatively healthy status as the outcome, as evaluated by self-reported chronic conditions, physical and cognitive function, and mental wellness, the observed association persisted (AOR, 1.54; 95% CI, 1.05-2.26). When adding the redefined alcohol use status, which better reflected the alcohol consumption amount, to the HLS-100, there was a slight increase in the estimated AOR (1.17; 95% CI, 1.11-1.24) per-unit increase in healthy lifestyle scores compared with HLS-100 (AOR, 1.13; 95% CI, 1.07-1.19). In addition, when excluding components from the healthy lifestyle score one at a time, acquiring the highest healthy lifestyle score was persistently associated with the survivorship of becoming a centenarian, which was comparable with our main results.

**Table 3.** Sensitivity Analysis for Association Between Healthy Lifestyle Score and Becoming a Centenarian

Measurement	Healthy lifestyle score^a^			Per unit increase
	Unfavorable	Intermediate	Favorable	
**Excluding cases (n = 268) who lived to be centenarians within 2 y and their matched controls (n = 268)**				
Cases, No./total No. (%)	298/1318 (22.6)	654/2584 (25.3)	234/784 (29.9)	NA
Odds ratio (95% CI)^b^	1 [Reference]	1.20 (1.02-1.42)	1.58 (1.28-1.96)	1.12 (1.06-1.18)
**Excluding cases (n = 813) who lived to be centenarians within 5 y and their matched controls (n = 1344)^c^**				
Cases, No./total No. (%)	144/792 (18.2)	350/1702 (20.6)	147/571 (25.7)	NA
Odds ratio (95% CI)^b^	1 [Reference]	1.17 (0.94-1.46)	1.55 (1.19-2.02)	1.12 (1.05-1.20)
**Redefining a standardized weighted healthy lifestyle score^d^**				
Cases, No./total No. (%)	204/881 (23.2)	1014/3623 (28.0)	236/718 (32.9)	NA
Odds ratio (95% CI)^b^	1 [Reference]	1.25 (1.03-1.51)	1.72 (1.36-2.18)	1.13 (1.08-1.19)
**Becoming a healthy aging centenarian as an outcome^e^**				
Cases, No./total No. (%)	124/1486 (8.3)	228/2885 (7.9)	76/851 (8.9)	NA
Odds ratio (95% CI)^b^	1 [Reference]	1.00 (0.75-1.32)	1.54 (1.05-2.26)	1.13 (1.02-1.25)
**Updated healthy lifestyle score^f^**				
Cases, No./total No. (%)	352/1363 (25.8)	595/1351 (44.0)	167/321 (52.0)	NA
Odds ratio (95% CI)^b^	1 [Reference]	2.12 (1.73-2.60)	3.66 (2.72-4.92)	1.37 (1.27-1.47)
**Cohort analysis**				
Cases, No./total No. (%)	576/3274 (17.6)	1229/5736 (21.4)	413/1768 (23.4)	NA
Odds ratio (95% CI)^b^	1 [Reference]	1.58 (0.99-2.50)	1.31 (0.72-2.39)	1.10 (0.94-1.28)
**Excluding participants with missing covariates (n = 429)**				
Cases, No./total No. (%)	331/1338 (24.7)	744/2663 (27.9)	254/792 (32.1)	NA
Odds ratio (95% CI)	1 [Reference]	1.23 (1.04-1.45)	1.63 (1.32-2.01)	1.12 (1.07-1.19)
**Redefining and including drinking status using alcohol consumption (g/day)^g^**				
Cases, No./total No. (%)	60/318 (18.9)	608/2423 (25.1)	310/1064 (29.1)	NA
Odds ratio (95% CI)^b^^,^^h^	1 [Reference]	1.55 (1.09-2.19)	2.11 (1.46-3.05)	1.17 (1.11-1.24)
**Excluding smoking status component^i^**				
Cases, No./total No. (%)	1078/4001 (26.9)	NA	376/1221 (30.8)	NA
Odds ratio (95% CI)^b^^,^^j^	1 [Reference]	NA	1.38 (1.18-1.60)	1.13 (1.06-1.19)
**Excluding exercise status component^i^**				
Cases, No./total No. (%)	2238/2049 (25.3)	NA	935/3173 (29.5)	NA
Odds ratio (95% CI)^b^^,^^j^	1 [Reference]	NA	1.26 (1.09-1.44)	1.13 (1.05-1.21)
**Excluding dietary diversity component^i^**				
Cases, No./total No. (%)	1064/3926 (27.1)	NA	390/1296 (30.1)	NA
Odds ratio (95% CI)^b^^,^^j^	1 [Reference]	NA	1.22 (1.06-1.41)	1.13 (1.07-1.21)

Abbreviation: NA, not applicable.

^a^ The lifestyle score ranges include 0 to 2 unfavorable, 3 to 4 intermediate (favorable for smoking, exercise, and dietary diversity components), and 6 to 7 favorable (6-8 for “Redefining and including drinking status using alcohol consumption” and smoking, exercise, and dietary diversity components).

^b^ Adjusted for residence (urban dwellers, rural dwellers, and missing), duration of education (0, 1-9, >9 years, and missing), marital status (in married, not married, and missing), hypertension (yes, no, and missing), diabetes (yes, no, and missing), cardiovascular disease (yes, no, and missing), cancer (yes, no, and missing), alcohol use status (never, former, and current), and body mass index (<18.5, 18.5-23.9, and ≥24.0 [calculated as weight in kilograms divided by height in meters squared]).

^c^ Median follow-up of the cases was approximately 5 (IQR, 3-7) years.

^d^ The standardized weighted healthy lifestyle score was redefined based on effect size of per-score increase in each lifestyle component.

^e^ Healthy aging centenarians were the centenarians with 3 or more of 4 healthy aging items, including no self-reported chronic conditions, normal physical function, normal cognitive function, and mental wellness. No self-reported chronic conditions: none of 4 chronic conditions, including hypertension, diabetes, cardiovascular disease, and cancer. Normal physical function: physical function was assessed using the Activities of Daily Living index, with physical function impairment defined as requiring any form of assistance (ranging from partial to complete help) in performing daily tasks, such as bathing, dressing, toileting, getting out of bed, and feeding. Normal cognitive function: a validated Chinese version of the Mini-Mental State Exam was used to test the cognitive function, with a score of 18 or higher indicating the absence of cognitive impairment. Mental wellness was defined as having no feeling of loneliness or anxiety.

^f^ The updated healthy lifestyle score was derived from the last measurement of smoking status, exercise status, and dietary diversity score, before the event.

^g^ Alcohol consumption status was calculated from beverage type and amount, assuming the follow alcohol content by volume (v/v) typically seen in China: strong liquor 53%, weak liquor 38%, grape wine 12%, rice wine 15%, and beer 4% (1-2). It was subsequently categorized as heavy (ie, >40 g/d for men, and >20 g/d for women; 0 point), moderate (ie, >0 and ≤40 g/d for men, and >0 and ≤20 g/d for women; 1 point), and never drinkers (0 point), and included in the healthy lifestyle score, after excluding cases (n = 476), and their matched controls and other controls (n = 941) who had missing alcohol consumption information (3).

^h^ Not adjusted for alcohol use status (never, former, and current).

^i^ Excluded lifestyle component was additionally adjusted as the covariate.

^j^ Additionally adjusted for the corresponding excluded lifestyle component.

## Discussion

In this large-scale, prospective nested case-control study, we observed that the overall healthy lifestyle score, which was based on 5 lifestyle aspects (smoking, alcohol use, exercise, dietary diversity, and BMI), was associated with the odds of becoming a centenarian in people aged 80 years or older. This association was not solely noted with any single component in the healthy lifestyle score and was not modified by sex, age, residence, educational background, or marital status.

As our outcome of becoming a centenarian and our study population with advanced age are unusual, it is difficult to directly compare with previous studies. Still, our findings are, in general, consistent with prior findings, indicating that healthier lifestyle is associated with lower mortality in older populations.^23,30,31^ More specifically, by targeting the older age group (≥80 years), this observed association between a higher HLS and a higher likelihood of becoming a centenarian extended our understanding that people with healthy lifestyle behaviors, even at a very advanced age, could still have better health outcomes compared with their counterparts, although in the present study, we lacked information on the lifestyle behaviors of the participants at their younger age and could not fully rule out the potential association between early exposure and their likelihood of becoming centenarians.

Among 5 classical lifestyle components, never smoking, currently exercising, and a more diverse diet stood out, as they were associated with survivorship to 100 years. Similar associations have been observed previously when inspecting single lifestyle factors and mortality risk,^23,31,32^ suggesting that these domains may be important targets for intervention. Although not supported by our data, previous findings suggested that moderate alcohol consumption was not necessarily related to adverse health outcomes^25,33^ and that higher BMI may have a protective role in mortality risk in older populations.^5,34^ When constructing an HLS-100 only including smoking status, exercise, and dietary diversity, we observed even greater odds in the association between healthy lifestyle behaviors and the likelihood of becoming centenarians, bringing up a critical question of whether the assessment of healthy lifestyle behaviors should be customized in different age groups. Specifically, BMI for individuals at very advanced ages may reflect potential malnutrition and other chronic conditions rather than being an indicator of lifestyle. In this case, defining an optimal BMI in older adults is a critical issue worth further investigation. In addition, studies are warranted to explore the underlying biological explanation for the observed association of smoking, exercise, and diet with the health outcomes of people at very advanced ages. An estimated 34.8% of the observed association between HLS-100 and the likelihood of living to age 100 years could be attributed to updated chronic conditions scoring according to the mediation analysis; however, this result should be interpreted with caution, as the statistical test did not find significant results.

Furthermore, in the series of sensitivity analyses, the robustness of our results was further confirmed by the 2- and 5-year lag test, 2 modified HLS-100 tools (a redefined standardized weighted HLS-100 and an updated HLS-100), restricting the outcome as becoming centenarians with a relatively healthy status, a cohort study design using Cox proportional hazards regression analysis, and excluding participants with incomplete covariate information. When redefined alcohol use status was further incorporated into HLS-100, the observed association persisted with slight increase in magnitude, suggesting that when additional information is available to capture alcohol consumption amount, it would be more appropriate to define alcohol use quantitatively rather than by drinking status (current, former, never). However, to gather that information, more comprehensive questionnaires are required, which could lead to a lower response rate and missing data. The rationale for the HLS we constructed was also supported by sensitivity analyses in which we excluded each factor.

### Strengths and Limitations

This study had the following strengths. The large sample size and thorough collection of lifestyle behaviors from this nationally representative survey of older Chinese individuals allowed us to prospectively evaluate the association between healthy lifestyle and becoming a centenarian in individuals of advanced age (≥80 years).

This study had several limitations. First, information on most lifestyle behaviors was self-reported and therefore subject to measurement error; specifically, exercise was referred to as organized leisure time activity, and the total energy expenditure from daily living cannot be fully captured. Second, medical conditions were self-reported; even with detailed explanations from trained field workers during the interview, it would inevitably underestimate the disease prevalence and lead to misclassification, also leaving residual confounding due to unmeasured medical conditions. Third, the lifestyle factors collected regarding the past lacked specificity (eg, in the past 1 year, 10 years), resulting in less accurate evaluation of the association between past lifestyle behaviors and health outcomes in people of very advanced age. Additionally, missing information on lifestyle factors in follow-up surveys limited our ability to keep track of lifestyle behaviors in a timely manner and calculate the updated lifestyle scores in the entire study population. Fourth, given the well-recognized correlation between lifestyle factors and socioeconomic status, even though we controlled for educational level, rural or urban residence, and marital status, residual confounding of unmeasured factors related to socioeconomic status, such as household income and occupation, cannot be fully ruled out. Fifth, although the present study was based on a nationwide cohort, the generalizability of our findings is limited due to about 26% of participants who were lost to follow-up, which may introduce selection bias. Sixth, even though the results from our lag analyses remained robust, the observational study setting does not allow inference of causality.

## Conclusions

In this nested case-control study of people aged 80 years or older in China, a healthier lifestyle score was associated with a higher likelihood of becoming a centenarian, underscoring the importance of adherence to a healthy lifestyle for better health outcomes even at very advanced ages. Developing appropriate intervention strategies targeting lifestyle improvement to promote health may be universally beneficial across different life stages.

## Notes

Supplement 1. **eMethods.** Detailed Methods
eReferences
**eTable 1.** Definitions of Lifestyle Components **eFigure.** Flow Chart of the Nested Case-Control Study **eTable 2.** Odds Ratios (ORs) and 95% Confidence Intervals (CIs) for Associations Between Five Lifestyle Components and Becoming a Centenarian **eTable 3.** The Distribution of the Number of Participants Across Healthy Lifestyle Score for 100 (HLS-100) **eTable 4.** The Predictive Probability of the Healthy Lifestyle Score for 100 (HLS-100) and Lifestyle Components for Becoming a Centenarian **eTable 5.** Association Between the Healthy Lifestyle Score for 100 (HLS-100, the Binary Version) and Likelihood of Becoming Centenarians **eTable 6.** Subgroup Analysis for Per Unit Increase of Healthy Lifestyle Score for 100 (HLS-100) and Becoming Centenarians **eTable 7.** The Potential Mediation Effect of Chronic Conditions on the Association Between HLS-100 and the Likelihood of Becoming Centenarians **eTable 8.** The β Coefficient and Weighted Score of Lifestyle Components for Redefining a Standardized Weighted Healthy Lifestyle Score

Supplement 2.
Data Sharing Statement
