# Supplementary material for: Healthy Lifestyle and the Likelihood of Becoming a Centenarian
Source: JAMA Netw Open. 2024 Jun 20;7(6):e2417931. doi: 10.1001/jamanetworkopen.2024.17931 (PMC11190803; doi:10.1001/jamanetworkopen.2024.17931)
Supplement: Supplement 1. — eMethods. Detailed Methods eReferences eTable 1. Definitions of Lifestyle Components eFigure. Flow Chart of the Nested Case-Control Study eTable 2. Odds Ratios (ORs) and 95% Confidence Intervals (CIs) for Associations Between Five Lifestyle Components and Becoming a Centenarian eTable 3. The Distribution of the Number of Participants Across Healthy Lifestyle Score for 100 (HLS-100) eTable 4. The Predictive Probability of the Healthy Lifestyle Score for 100 (HLS-100) and Lifestyle Components for Becoming a Centenarian eTable 5. Association Between the Healthy Lifestyle Score for 100 (HLS-100, the Binary Version) and Likelihood of Becoming Centenarians eTable 6. Subgroup Analysis for Per Unit Increase of Healthy Lifestyle Score for 100 (HLS-100) and Becoming Centenarians eTable 7. The Potential Mediation Effect of Chronic Conditions on the Association Between HLS-100 and the Likelihood of Becoming Centenarians eTable 8. The β Coefficient and Weighted Score of Lifestyle Components for Redefining a Standardized Weighted Healthy Lifestyle Score [file jamanetwopen-e2417931-s001.pdf]

# Supplementary Online Content

Li Y, Wang K, Jigeer G, et al. Healthy lifestyle and the likelihood of becoming a centenarian: findings from the Chinese Longitudinal Healthy Longevity Survey. *JAMA Netw Open*. 2024;7(6):e2417931. doi:10.1001/jamanetworkopen.2024.17931

**eMethods.** Detailed Methods

**eReferences**

**eTable 1.** Definitions of Lifestyle Components

**eFigure.** Flow Chart of the Nested Case-Control Study

**eTable 2.** Odds Ratios (ORs) and 95% Confidence Intervals (CIs) for Associations Between Five Lifestyle Components and Becoming a Centenarian

**eTable 3.** The Distribution of the Number of Participants Across Healthy Lifestyle Score for 100 (HLS-100)

**eTable 4.** The Predictive Probability of the Healthy Lifestyle Score for 100 (HLS-100) and Lifestyle Components for Becoming a Centenarian

**eTable 5.** Association Between the Healthy Lifestyle Score for 100 (HLS-100, the Binary Version) and Likelihood of Becoming Centenarians

**eTable 6.** Subgroup Analysis for Per Unit Increase of Healthy Lifestyle Score for 100 (HLS-100) and Becoming Centenarians

**eTable 7.** The Potential Mediation Effect of Chronic Conditions on the Association Between HLS-100 and the Likelihood of Becoming Centenarians

**eTable 8.** The β Coefficient and Weighted Score of Lifestyle Components for Redefining a Standardized Weighted Healthy Lifestyle Score

This supplementary material has been provided by the authors to give readers additional information about their work.

© 2024 Li Y et al. *JAMA Network Open.*

**eMethods. Detailed Methods**

**Assessment of individual lifestyle factors**

Specifically, the status of smoking (never: 2, former: 1, and current: 0) was determined using two yes-or-no questions: "Do you smoke cigarettes at present?", and "Did you smoke cigarettes in the past?". People who answered yes for the first questions were defined as current smokers regardless of the answer for the second question; people who answered no for the first question but yes for the second question were categorized as former smokers, otherwise as never smokers. Likewise, the status of alcohol use (never: 2, former: 1, and current: 0), and exercise (current: 2, former: 1, and never: 0) were determined similarly by the following questions: "Do you drink alcohol at present?", and "Did you drink alcohol in the past?". "Do you exercise regularly (eg. Running, Qigong, etc.) at present?", and "Did you exercise regularly (eg. Running, Qigong, etc.) in the past?" [1] Dietary intake was evaluated based on a food frequency questionnaire, [2] and dietary diversity was assessed according to the frequency of consuming five food groups: fruits, vegetables, fish, beans, and tea. Participants reported "almost every day," "except winter or sometimes or occasionally," or "rarely or never." for the intake frequency of each food group, and were assigned scores of 2, 1, or 0 accordingly, [3] generating a total dietary diversity score ranged from 0 to 10. We then classified scores of 7-10 as favorable (2 points), 4-6 as intermediate (1 point), and 0-3 as unfavorable (0 point) for inclusion in the healthy lifestyle score calculation (**eTable 1 in the Supplement**). Given that height measurements were unavailable in the first four waves of surveys, knee height, measured by trained medical staff during the physical examination, was used to estimate individual height, following two validated equations focused on older Chinese, developed by Zhang et al. (Men: height=67.78+2.01×knee height; women: height=74.08+1.81×knee height). [4] BMI was calculated as measured weight (kg) /estimated height squared ($m^2$), and categorized as underweight (BMI < 18.5

© 2024 Li Y et al. *JAMA Network Open.*

kg/m$^2$), normal (18.5 kg/m$^2$ ≤ BMI < 24.0 kg/m$^2$), overweight/obese (BMI ≥ 24.0 kg/m$^2$).[5] As underweight has been associated with increased risk of all-cause mortality while overweight was inversely associated with mortality among people ≥ 80 years old in China,[6] we assigned scores of 0, 1, 2 for the underweight, overweight/obese, and normal weight participants.

**Redefined drinking status**

Alcohol consumption amount was used to redefine drinking status[7,8], determined by two additional questions: "How much alcohol per day on average do you drink at present, or in the past (if quit drinking)" and "What kind of alcohol do you drink at present, or in the past (if quit drinking)", categorized individuals as heavy (i.e. ≥41 g/day for men, and ≥21g/day for women; 0 points), moderate (i.e. >0 and ≤40 g/day for men, and >0 and ≤20g/day for women; 1 point), or none drinkers (0 g/day; 2 points), according to WHO's International guide for monitoring alcohol consumption and related harm[9]. This 3-point scale for alcohol consumption was then incorporated into the HLS-100.

**Assessment of physical and cognitive function, and mental wellness**

Physical function was assessed using the Activities of Daily Living (ADL) index, with normal physical function defined as not requiring any form of assistance (ranging from partial to complete help) in performing daily tasks such as bathing, dressing, toileting, getting out of bed, and feeding.[10] Cognitive function was evaluated by a validated Chinese version of the Mini-Mental State Exam (MMSE), with a score ≥18 indicating normal cognitive performance.[11] Mental wellness was determined by two questions asking about the feeling of loneliness and anxiety: "How often do you feel lonely or isolated" and "How often do you feel anxious", participants who answered "sometimes", "often", or "always" were classified as "yes" and participants who answered "never" or "seldom" were classified as "no" for each question, and having no feeling of loneliness or

© 2024 Li Y et al. *JAMA Network Open.*

anxiety were considered as good mental wellness.

**Association between healthy lifestyle and likelihood of becoming centenarians using the binary version HLS-100**

We conducted a secondary analysis using the three components as for HLS-100, in which each lifestyle factor was categorized as binary variables, never smoker, current exercisers, and individuals with favorable diet diversity score received 1 point, respectively, otherwise received 0 points, generating a binary version HLS-100 ranging from 0-3 points, then we categorized individuals with 0-1 points as unhealthy lifestyle group, and those with 2-3 points as healthy lifestyle group. With the binary version HLS-100, we re-examined the association between healthy lifestyle and likelihood of becoming centenarians.

**Estimation of predictive possibility of becoming centenarians**

We developed a predictive model to estimate the possibility of becoming centenarians at individual level based on the aforementioned variables, [12] then computed the group average according to different HLS-100 category and scores of individual healthy lifestyle factors (smoking, exercise, and dietary diversity).

**Subgroup analysis**

Interactions between HLS-100 and residence, years of education, marital status, chronic conditions, alcohol use status, and BMI were examined with the likelihood ratio test, and subgroup analyses were conducted when significant interaction was observed.

**Mediation analysis**

We further conducted a mediation analysis using the most recent chronic conditions before the endpoint event (death for controls and living to 100 years for cases) to examine the potential mediation effect of chronic conditions on the association between HLS-100 and the likelihood of

© 2024 Li Y et al. *JAMA Network Open.*

becoming centenarians.

**eReferences**


1. Sun C, Liu H, Xu F, et al. Combined lifestyle factors on mortality among the elder population: evidence from a Chinese cohort study. *BMC Geriatr.* 2022;22(1):474.
2. Yin Z, Fei Z, Qiu C, et al. Dietary Diversity and Cognitive Function among Elderly People: A Population-Based Study. *J Nutr Health Aging.* 2017;21(10):1089-1094.
3. Jin X, He W, Zhang Y, et al. Association of APOE epsilon4 genotype and lifestyle with cognitive function among Chinese adults aged 80 years and older: A cross-sectional study. *PLoS Med.* 2021;18(6):e1003597.
4. Zhang H, Hsu-Hage BH, Wahlqvist ML. The use of knee height to estimate maximum stature in elderly Chinese. *J Nutr Health Aging.* 1998;2(2):84-87.
5. Chen C, Lu FC, Department of Disease Control Ministry of Health PRC. The guidelines for prevention and control of overweight and obesity in Chinese adults. *Biomed Environ Sci.* 2004;17 Suppl:1-36.
6. Wang J, Taylor AW, Zhang T, Appleton S, Shi Z. Association between Body Mass Index and All-Cause Mortality among Oldest Old Chinese. *J Nutr Health Aging.* 2018;22(2):262-268.
7. Millwood IY, Li L, Smith M, et al. Alcohol consumption in 0.5 million people from 10 diverse regions of China: prevalence, patterns and socio-demographic and health-related correlates. *Int J Epidemiol.* 2013;42(3):816-827.
8. Li J, Wu B, Tevik K, Krokstad S, Helvik AS. Factors associated with elevated consumption of alcohol in older adults-comparison between China and Norway: the CLHLS and the HUNT Study. *BMJ Open.* 2019;9(8):e028646.


© 2024 Li Y et al. *JAMA Network Open.*

9. World Health Organization. (2000). International guide for monitoring alcohol consumption and related harm. World Health Organization. https://iris.who.int/handle/10665/66529.

10. Katz S, Ford AB, Moskowitz RW, Jackson BA, Jaffe MW. STUDIES OF ILLNESS IN THE AGED. THE INDEX OF ADL: A STANDARDIZED MEASURE OF BIOLOGICAL AND PSYCHOSOCIAL FUNCTION. *Jama.* 1963;185:914-919.

11. Gao M, Kuang W, Qiu P, Wang H, Lv X, Yang M. The time trends of cognitive impairment incidence among older Chinese people in the community: based on the CLHLS cohorts from 1998 to 2014. *Age Ageing.* 2017;46(5):787-793.

12. Iasonos A, Schrag D, Raj GV, Panageas KS. How to build and interpret a nomogram for cancer prognosis. *J Clin Oncol.* 2008;26(8):1364-1370.

© 2024 Li Y et al. *JAMA Network Open.*

**eTable 1. Definitions of lifestyle components.**

| Lifestyle components | Items: scores | | |
|---|---|---|---|
| Smoking status | Never: 2 | Former: 1 | Current: 0 |
| Drinking status | Never: 2 | Former: 1 | Current: 0 |
| Exercise status | Current: 2 | Former: 1 | Never: 0 |
| Dietary diversity score [a] | Favorable: 2 | Intermediate: 1 | Unfavorable: 0 |
| Body mass index (BMI), $kg/m^2$ | 18.5-23.9 (normal weight): 2 | ≥ 24.0 (overweight/obese): 1 | < 18.5 (underweight): 0 |

[a] Dietary diversity was evaluated based on the frequency of consuming seven food groups: fruits, vegetables, fish, beans, and tea. Participants reporting "almost every day," "except winter or sometimes or occasionally," or "rarely or never" for consuming each food item were separately assigned scores of 2, 1, or 0, and the total score of dietary diversity ranged from 0 to 10. Then, scores of 7-10 were classified as favorable=2, 4-6 as intermediate=1, and 0-3 as unfavorable=0.

© 2024 Li Y et al. *JAMA Network Open.*

**eFigure. Flow chart of the nested case-control study.**

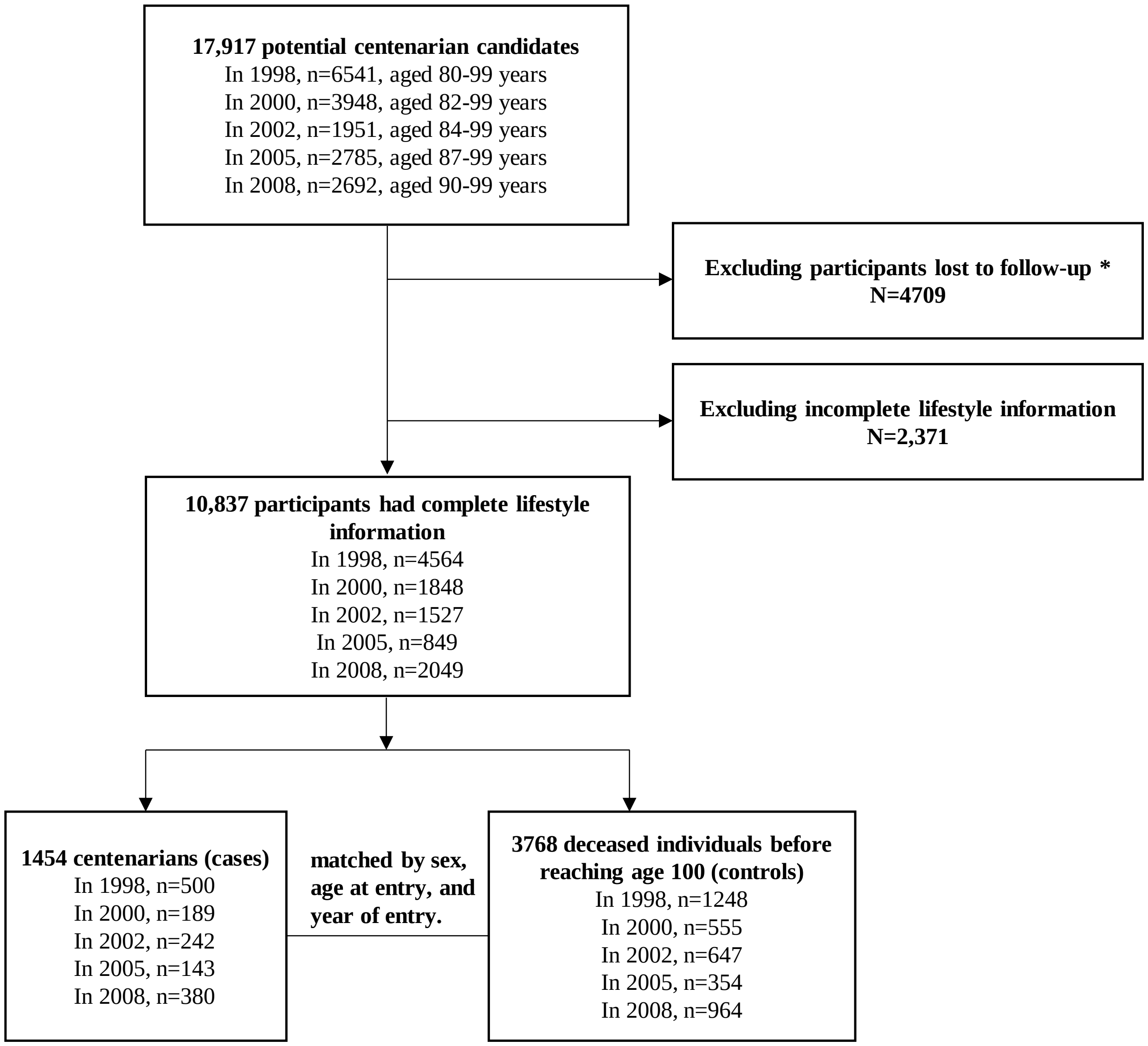


* Loss to follow-up was defined as participants becoming unreachable after at least three attempts to contact.

© 2024 Li Y et al. *JAMA Network Open.*

**eTable 2. Odds ratios (ORs) and 95% confidence intervals (CIs) for associations between five lifestyle components and becoming a centenarian.**

| | | | | |
|---|---|---|---|---|
| **Smoking status** | **Current (unfavorable)** | **Former (intermediate)** | **Never (favorable)** | **Per unit increase** |
| **Cases / Total (%)** | 194 / 787 (24.7) | 160 / 677 (23.6) | 1100 / 3758 (29.3) | NA |
| **OR (95% CI) [a]** | Reference | 0.94 (0.73, 1.22) | 1.25 (1.02, 1.53) | 1.14 (1.03, 1.26) |
| **Alcohol use status** | **Current (unfavorable)** | **Former (intermediate)** | **Never (favorable)** | **Per unit increase** |
| **Cases / Total (%)** | 295 / 1047 (28.2) | 129 / 482 (26.8) | 1030 / 3693 (27.9) | NA |
| **OR (95% CI) [a]** | Reference | 0.97 (0.75, 1.25) | 0.95 (0.80, 1.12) | 0.98 (0.90, 1.06) |
| **Exercise status** | **Never (unfavorable)** | **Former (intermediate)** | **Current (favorable)** | **Per unit increase** |
| **Cases / Total (%)** | 966 / 3541 (27.3) | 91 / 388 (23.5) | 397 / 1293 (30.7) | NA |
| **OR (95% CI) [a]** | Reference | 0.80 (0.61, 1.04) | 1.31 (1.13, 1.53) | 1.13 (1.05, 1.22) |
| **Dietary diversity score [b]** | **0-3 (unfavorable)** | **4-6 (intermediate)** | **7-10 (favorable)** | **Per unit increase** |
| **Cases / Total (%)** | 275 / 1014 (27.1) | 863 / 3631 (27.6) | 316 / 1086 (29.1) | NA |
| **OR (95% CI) [a]** | Reference | 1.09 (0.92, 1.29) | 1.23 (1.00, 1.52) | 1.11 (1.00, 1.23) |
| **Body mass index (BMI), kg/m$^2$** | **<18.5 (unfavorable)** | **≥ 24.0 (intermediate)** | **18.5-23.9 (favorable)** | **Per unit increase** |
| **Cases / Total (%)** | 711 / 2553 (27.9) | 161 / 592 (27.2) | 582 / 2077 (28.0) | NA |
| **OR (95% CI) [a]** | Reference | 1.09 (0.88, 1.36) | 1.10 (0.95, 1.26) | 1.05 (0.98, 1.12) |

[a] Adjusted for residence (urban dwellers, rural dwellers, and missing), duration of education (0, 1-9, > 9 years, and missing), marital status (in marriage, not in marriage, and missing), hypertension (yes, no, and missing), diabetes (yes, no, and missing), cardiovascular disease (CVD) (yes, no, and missing), cancer (yes, no, and missing). The five lifestyle components mutually adjusted for each other.

[b] Dietary diversity was evaluated based on the frequency of consuming seven food groups: fruits, vegetables, fish, beans, and tea. Participants reporting “almost every day,” “except winter or sometimes or occasionally,” or “rarely or never” for consuming each food item were separately assigned scores of 2, 1, or 0, and the total score of dietary diversity ranged from 0 to 10. Then, scores of 7-10 were classified as favorable=2, 4-6 as intermediate=1, and 0-3 as unfavorable=0.

© 2024 Li Y et al. *JAMA Network Open.*

**eTable 3. The distribution of the number of participants across healthy lifestyle score for 100 (HLS-100).**

| Healthy lifestyle score | Case/Total (%) |
|---|---|
| **0** | 23 / 100 (23.0) |
| **1** | 91 / 370 (24.6) |
| **2** | 259 / 1016 (25.5) |
| **3** | 550 / 2002 (27.5) |
| **4** | 255 / 883 (28.9) |
| **5** | 195 / 585 (33.3) |
| **6** | 81 / 266 (30.5) |

© 2024 Li Y et al. *JAMA Network Open.*

**eTable 4. The predictive probability of the healthy lifestyle score for 100 (HLS-100) and lifestyle components for becoming a centenarian.**

| **HLS-100** | **0-2 (unfavorable)** | **3-4 (intermediate)** | **5-6 (favorable)** | **Per unit increase** |
|---|---|---|---|---|
| Average predictive probability (95% CI, %) [a] | 16.9 (15.0, 18.8) | 19.9 (18.0, 21.8) | 26.3 (24.5, 28.2) | 3.15 (3.08, 3.23) |
| Difference of predictive probability (95% CI, %) [a, b] | NA | 2.98 (2.82 3.15) | 9.44 (9.67, 9.22) | NA |
| **Smoking status** | **Current (unfavorable)** | **Former (intermediate)** | **Never (favorable)** | **Per unit increase** |
| Average predictive probability (95% CI, %) [a] | 18.4 (16.3, 20.4) | 19.5 (17.4, 21.5) | 21.9 (19.8, 23.9) | 1.82 (1.70, 1.94) |
| Difference of predictive probability (95% CI, %) [a, b] | NA | 1.06 (0.768, 1.36) | 3.56 (3.33, 3.80) | NA |
| **Exercise status** | **Never (unfavorable)** | **Former (intermediate)** | **Current (favorable)** | **Per unit increase** |
| Average predictive probability (95% CI, %) [a] | 17.6 (15.5, 19.6) | 19.5 (17.4, 21.6) | 22.6 (20.6, 24.7) | 2.49 (2.39, 2.58) |
| Difference of predictive probability (95% CI, %) [a, b] | NA | 1.91 (2.21, 1.60) | 5.07 (4.88, 5.26) | NA |
| **Dietary diversity score [c]** | **0-3 (unfavorable)** | **4-6 (intermediate)** | **7-10 (favorable)** | **Per unit increase** |
| Average predictive probability (95% CI, %) [a] | 17.9 (15.8, 20.0) | 20.5 (18.4, 22.5) | 21.4 (19.3, 23.4) | 1.71 (1.58, 1.84) |
| Difference of predictive probability (95% CI, %) [a, b] | NA | 2.56 (2.77, 2.36) | 3.46 (3.20, 3.71) | NA |

[a] Adjusted for age (continuous), sex (men and women), entry year (1998, 2000, 2002, 2005, and 2008), residence (urban dwellers, rural dwellers, and missing), duration of education (0, 1-9, > 9 years, and missing), marital status (in marriage, not in marriage, and missing), hypertension (yes, no, and missing), diabetes (yes, no, and missing), cardiovascular disease (CVD) (yes, no, and missing), cancer (yes, no, and missing), alcohol use status (never, former, and current), and body mass index (<18.5 kg/m$^2$, 18.5-23.9 kg/m$^2$, and ≥24.0 kg/m$^2$)..

[b] Reference was the corresponding unfavorable group.

© 2024 Li Y et al. *JAMA Network Open.*

**eTable 5. Association between healthy lifestyle score for 100 (HLS-100, the binary version) and likelihood of becoming centenarians.**

| | | 0-1 (unfavorable) | 2-3 (favorable) | Per unit increase |
|---|---|---|---|---|
| **Lifestyle components as a binary variable** | Cases / Total (%) | 996 / 3783 (26.3) | 458 / 1439 (31.8) | NA |
| | OR (95% CI) [a] | Reference | 1.45 (1.27, 1.67) | 1.26 (1.16, 1.38) |

[a] Adjusted for residence (urban dwellers, rural dwellers, and missing), duration of education (0, 1-9, > 9 years, and missing), marital status (in marriage, not in marriage, and missing), hypertension (yes, no, and missing), diabetes (yes, no, and missing), cardiovascular disease (CVD) (yes, no, and missing), cancer (yes, no, and missing), alcohol use status (never, former, and current), and body mass index (<18.5 kg/$m^2$, 18.5-23.9 kg/$m^2$, and ≥24.0 kg/$m^2$).

© 2024 Li Y et al. *JAMA Network Open.*

**eTable 6. Subgroup analysis for per unit increase of healthy lifestyle score for 100 (HLS-100) and becoming centenarians.**

| | Cases /Total (%) | OR (95% CI) | *P* for interaction |
|---|---|---|---|
| **Residence** [a] | | | 0.64 |
| Urban dwellers | 890 / 3253 (27.4) | 1.13 (1.06, 1.20) | |
| Rural dwellers | 562 / 1960 (28.7) | 1.14 (1.05, 1.23) | |
| **Years of schooling** [a] | | | 0.23 |
| 0 | 1062 / 3725 (28.5) | 1.06 (0.99, 1.12) | |
| >0 | 388 / 1483 (26.2) | 1.31 (1.20, 1.43) | |
| **Married status** [a] | | | 0.08 |
| In a relationship | 177 / 718 (24.7) | 1.21 (1.06, 1.37) | |
| Not in a relationship | 1277 / 4504 (28.4) | 1.11 (1.06, 1.18) | |
| **Chronic conditions** [a] | | | 0.71 |
| Yes | 219 / 835 (26.2) | 1.39 (1.07, 1.80) | |
| No | 1180 / 4184 (28.2) | 1.25 (1.12, 1.40) | |
| **Alcohol use status** [a, b] | | | 0.32 |
| Current | 295 / 1063 (27.8) | 1.32 (1.06, 1.63) | |
| Non-current | 1159 / 4159 (27.9) | 1.25 (1.12, 1.40) | |
| **Body mass index** [a, c] | | | 0.28 |
| Normal weight (18.5-23.9 kg/$m^2$) | 582 / 2118 (27.5) | 1.27 (1.09, 1.49) | |
| Underweight or overweight/obese (<18.5 or ≥24.0 kg/$m^2$) | 872 / 3104 (28.1) | 1.27 (1.12, 1.44) | |

[a] Adjusted for residence (urban dwellers, rural dwellers, and missing), duration of education (0, 1-9, > 9 years, and missing), marital status (in marriage, not in marriage, and missing), hypertension (yes, no, and missing), diabetes (yes, no, and missing), cardiovascular disease (CVD) (yes, no, and missing), cancer (yes, no, and missing), alcohol use status (never, former, and current), and body mass index (<18.5 kg/$m^2$, 18.5-23.9 kg/$m^2$, and ≥24.0 kg/$m^2$).

[b] Not adjusted for alcohol use status (never, former, and current).

[c] Not adjusted for body mass index (<18.5 kg/$m^2$, 18.5-23.9 kg/$m^2$, and ≥24.0 kg/$m^2$).

© 2024 Li Y et al. *JAMA Network Open.*

**eTable 7. The potential mediation effect of chronic conditions on the association between HLS-100 and the likelihood of becoming centenarians.**

| **Potential mediators** | | **β (95% CI)** [b] | ***P*** |
|---|---|---|---|
| **Updated chronic conditions** [a] | Direct Effect | 0.114 (-0.044, 0.271) | 0.16 |
| | Indirect Effect | 0.056 (0.005, 0.107) | 0.03 |
| | Total effect | 0.169 (0.003, 0.335) | 0.045 |
| | Mediation Effect Proportion | 34.8% (-1.9%, 71.5%) | 0.06 |

[a] Updated chronic conditions: the most recent chronic conditions before the endpoint event (death for controls and living to 100 years for cases).

[b] Adjusted for age (continuous), sex (men and women), entry year (1998, 2000, 2002, 2005, and 2008), residence (urban dwellers, rural dwellers, and missing), duration of education (0, 1-9, > 9 years, and missing), marital status (in marriage, not in marriage, and missing), hypertension (yes, no, and missing), diabetes (yes, no, and missing), cardiovascular disease (CVD) (yes, no, and missing), cancer (yes, no, and missing), alcohol use status (never, former, and current), and body mass index (<18.5 $kg/m^2$, 18.5-23.9 $kg/m^2$, and ≥24.0 $kg/m^2$).

© 2024 Li Y et al. *JAMA Network Open.*

**eTable 8. The β coefficient and weighted score of lifestyle components for redefining a standardized weighted healthy lifestyle score.**

| **Components** | **β** | **Weighted score** | | |
|---|---|---|---|---|
| | | **Unfavorable** | **Intermediate** | **Favorable** |
| Smoking status | 0.1262 | 0 | 1.04 | 2.08 |
| Exercise status | 0.1248 | 0 | 1.03 | 2.06 |
| Dietary diversity score | 0.1122 | 0 | 0.93 | 1.85 |

© 2024 Li Y et al. *JAMA Network Open.*
