# Supplementary material for: Healthy Lifestyle and the Likelihood of Becoming a Centenarian
Source: JAMA Netw Open. 2024 Jun 20;7(6):e2417931. doi: 10.1001/jamanetworkopen.2024.17931 (PMC11190803; doi:10.1001/jamanetworkopen.2024.17931)
Supplement: Supplement 2. — Data Sharing Statement [file jamanetwopen-e2417931-s002.pdf]

Data Sharing Statement

Li. Healthy Lifestyle and the Likelihood of Becoming a Centenarian. *JAMA Netw Open*. Published June 20, 2024. doi:10.1001/jamanetworkopen.2024.17931

Data
Data available: Yes
Data types: Deidentified participant data
How to access data: All data requests should be submitted to the corresponding author (XG: xiang_gao@fudan.edu.cn) for consideration. Access to anonymized data may be granted upon reasonable request.
When available: With publication

Supporting Documents
Document types: None

Additional Information
Who can access the data: Researchers whose proposed use of the data has been approved
Types of analyses: For a specified purpose
Mechanisms of data availability: After approval of a proposal
